# Evaluation of the Microstructure, Tribological Characteristics, and Crack Behavior of a Chromium Carbide Coating Fabricated on Gray Cast Iron by Pulsed-Plasma Deposition

**DOI:** 10.3390/ma14123400

**Published:** 2021-06-19

**Authors:** Yuliia Chabak, Vasily Efremenko, Miroslav Džupon, Kazumichi Shimizu, Victor Fedun, Kaiming Wu, Bohdan Efremenko, Ivan Petryshynets, Tatiana Pastukhova

**Affiliations:** 1 Physics Department, Pryazovskyi State Technical University, 87555 Mariupol, Ukraine; julia.chabak25@gmail.com (Y.C.); fedun276@gmail.com (V.F.); bodyaefr@gmail.com (B.E.); tetianapast@gmail.com (T.P.); 2 Institute of Materials Research, Slovak Academy of Sciences, 04001 Kosice, Slovakia; mdzupon@saske.sk (M.D.); ipetryshynets@saske.sk (I.P.); 3 Mechanical Engineering Research Unit, College of Design and Manufacturing Technology, Muroran Institute of Technology, Muroran 050-8585, Japan; shimizu@mmm.muroran-it.ac.jp; 4 The State Key Laboratory of Refractories and Metallurgy, Hubei Province Key Laboratory of Systems Science in Metallurgical Process, Collaborative Innovation Center for Advanced Steels, International Research Institute for Steel Technology, Wuhan University of Science and Technology, Wuhan 430081, China; wukaiming@wust.edu.cn

**Keywords:** pulsed-plasma deposition, protective coating, carbide, wear resistance, cracks

## Abstract

The structural and tribological properties of a protective high-chromium coating synthesized on gray cast iron by air pulse-plasma treatments were investigated. The coating was fabricated in an electrothermal axial plasma accelerator equipped with an expandable cathode made of white cast iron (2.3 wt.% C–27.4 wt.% Cr–3.1 wt.% Mn). Optical microscopy, scanning electron microscopy, energy-dispersive spectroscopy, X-ray diffraction analysis, microhardness measurements, and tribological tests were conducted for coating characterizations. It was found that after ten plasma pulses (under a discharge voltage of 4 kV) and post-plasma heat treatment (two hours of holding at 950 °C and oil-quenching), a coating (thickness = 210–250 µm) consisting of 48 vol.% Cr-rich carbides (M_7_C_3_, M_3_C), 48 vol.% martensite, and 4 vol.% retained austenite was formed. The microhardness of the coating ranged between 980 and 1180 HV. The above processes caused a gradient in alloying elements in the coating and the substrate due to the counter diffusion of C, Cr, and Mn atoms during post-plasma heat treatments and led to the formation of a transitional layer and different structural zones in near-surface layers of cast iron. As compared to gray cast iron (non-heat-treated and heat-treated), the coating had 3.0–3.2 times higher abrasive wear resistance and 1.2–1208.8 times higher dry-sliding wear resistance (depending on the counter-body material). The coating manifested a tendency of solidification cracking caused by tensile stress due to the formation of a mostly austenitic structure with a lower specific volume. Cracks facilitated abrasive wear and promoted surface spalling under dry-sliding against the diamond cone.

## 1. Introduction

Gray cast iron (GCI) is a traditional structural material that is widely used in mechanical engineering due to its good manufacturability, low production cost, improved vibro-damping properties, and better dry-sliding wear resistance [1]. However, GCI is characterized by its low abrasive wear resistance because of the presence of the graphite phase, which easily degrades under abrasion [2]. This common problem can be solved by surface modification or protective coating deposition. Laser beam scanning is an effective approach to structurally modify GCI and increase its abrasive wear resistance [3]. Pang et al. [4], Sui et al. [5,6], and Yang et al. [7] applied a laser Nd:YAG treatment to form biomemically located modified areas on the GCI surface. The modification was achieved without changing the chemical composition of GCI and by forming chilled areas with a hard ledeburite structure under rapid crystallization after laser melting. The matrix of the chilled areas consisted of martensite and retained metastable austenite, which contributed to an improved wear resistance [8]. Different techniques, such as surface doping in the mold [9], laser carburizing [10], and direct surface alloying through the deep laser beam melting of preliminary deposited carbides (Cr_3_C_2_, WC) [11,12], have been adopted to create the composite structure on GCI surface. Zhong et al. [13] revealed the improvement of corrosion and wear characteristics of gray iron liner resulted from laser alloying by Ni-Cr powder. A similar result was obtained in [14], where Cr laser alloying was used to enhance GCI thermal fatigue resistance. Laser cladding of Fe-based alloy [15] and Ni-based alloy [16] was applied to a chemically modified gray cast aiming at voids elimination and further interfacial behaviors regulation.

The other direction of GCI properties improvement is a protective coating deposition. Different techniques were repeatedly applied to deposit the coating on the GCI’s surface. They are powder thermal spraying [17], arc spraying/sintering [18], physical vapor deposition [19,20,21], laser cladding [22,23], plasma surfacing [24,25,26], plasma spraying [27,28], electric contact deposition [29], etc., which were previously reported to improve GCI’s resistance to abrasive wear, corrosion, thermal fatigue, and cavitation damage.

Among the variety of surface treatment techniques, the plasma-related methods stand out due to their advantages for surface modification and coating deposition. Sokolov et al. [30] and Park et al. [31] applied plasma ionic nitriding to enhance the corrosion resistance and cavitation resistance of GCI. Agarwal et al. [32] employed plasma nitrocarburization to improve the tribological properties of stamping dies made of GCI. Moreover, plasma electrolyte oxidation and ceramic coating deposition have also been used for GCI surface modification [33,34]. Cheng et al. [35] improved the wear characteristics of gray nodular cast iron by steady plasma beam surface melting. The detonation-based pulsed-plasma treatment proposed by Tyurin et al. [36,37] was also used to modify ductile cast iron [38]. Cherenda et al. [39] reported the application of a compression plasma flow to form a coating of the GCI surface. Kolyada et al. [40,41] highlighted the applicability of high-dynamic pulsed plasma flow generated in an electrothermal axial plasma accelerator (EAPA) for metallic surface modification. The design and operational principles of EAPA, which operates under atmospheric conditions, are outlined in detail in earlier studies [40,41,42]. Furthermore, EAPA was used for the surface structure modification (case hardening) of steel [43] and GCI [44]. Moreover, EAPA was employed for the deposition of coatings due to the erosion of the axial electrode (cathode) under high-current discharge [45,46]. However, EAPA was not previously attempted to deposit the protective coating on the GCI surface. Assuming the graphite presence in GCI, the coating/substrate interaction with carbon diffusion to the coating was expected to affect the coating performance. Considering the novelty of this approach, the present work was aimed at studying microstructural and tribological properties of a high-chromium (28 wt.%) coating deposited on the surface of GCI by EAPA pulsed-plasma deposition.

## 2. Materials and Methods

The substrate for the coating was made of gray cast iron with a chemical composition of 2.95 wt.% C, 1.45 wt.% Si, 0.95 wt.% Mn, 0.11 wt.% P, and 0.08 wt.% S. Specimens of 6 mm × 12 mm × 25 mm size were cut from a casting of 30 mm diameter and 300 mm length. The source material for coating deposition was high-Cr white cast iron with a chemical composition of 2.34 wt.% C, 27.39 wt.% Cr, 3.13 wt.% Mn, 1.26 wt.% Si, 0.20 wt.% Ti. A high-chromium cast iron rod of 5 mm diameter served as the cathode of a plasma accelerator.

Pulsed-plasma deposition was performed on an electrothermal axial plasma accelerator. EAPA was an arrester in the form of a paper-reinforced bakelite tube of 430 mm length, 8 mm inner diameter, and 17 mm wall thickness [42]. It was connected to an electrical circuit consisting of a capacitive energy storage device of 1.5 mF capacity [40]. A pulsed arc discharge with a current of ≈18 kA and a voltage of ≈4.5 kV was initiated inside a narrow dielectric channel between the axially positioned cathode (high-Cr cast iron) and the anode (end steel flange), leading to a rapid pressure increase in the channel (up to 100–150 atm) with the formation and injection of the plasma flow [43]. Under arc discharging, the evaporation/melting of inner walls (paper-reinforced bakelite) and the electrodes took place; thus, the products of EAPA erosion (ions, microdroplets) were taken away by plasma flow to form a coating on the target surface [44,45].

Pulsed-plasma deposition was performed in the air with the following parameters: voltage stored in the capacitor = 4.0 kV, distance between EAPA electrodes = 50 mm, distance from the EAPA edge to the target surface = 50 mm, pulse number = 10. The as-deposited specimens were subjected to a post-plasma heat treatment (two hours of holding at 950 °C) followed by oilcooling [42,46]. During heating, the coating was covered by coal to prevent surface oxidation and decarburization.

Furthermore, for microstructural characterizations, transversely cross-sectioned specimens were prepared by polishing using SiC sandpapers and alumina aqueous solutions to get a mirror state and etched in 4% nital solution. An optical microscope Eclipse M200 (Nikon, Tokyo, Japan) and a scanning electron microscope JSM-6510 (JEOL, Tokyo, Japan) were used for microstructural observations. The phase chemical composition was detected by an energy-dispersive spectrometer (EDS) equipped with a JED-2300 detector (JEOL, Tokyo, Japan). The phase composition of the coating was revealed by an X’Pert PRO diffractometer (PANalytical, Malvern, UK) under Cu-K_α_ radiation and the following parameters: the generator voltage is 40 kV, the tube current is 50 mA, the scan step is 0.03342 degrees, and the scan speed is 0.0689 degree/s. The volume fraction of retained austenite was calculated as [47]:

(1) $$VF_{A}=\frac{100\%}{1+0.72(I_{\alpha }/I_{\gamma })}$$

Microhardnesstests were performed by an FM-300microhardness tester (Future-Tech Corp., Kawasaki, Japan) under a load of 20 g. SEM images were used to measurecarbide size and carbide volume fraction (by the lineal Rozival method [48]).

The tribological characteristics of the coating were evaluated by “Three-body abrasion” tests and “Ball-on-plate” dry-sliding tests at room temperature under a relative humidity of 60–70% controlled by humidity sensor head Testo 625. Before abrasive wear testing, the surface of gray cast iron was ground by sandpaper to R_a_ = 0.638 μm and R_z_ = 2.387 μm; the coating surface had an as-coated condition. During “Three-body abrasion” tests, the specimen was pressed to a rubber roller of 40 mm diameter under a load of 20 N at a rotating speed of 10.8 s^−1^. Abrasive particles (Al_2_O_3_ of 0.5–0.6 mm diameter) were fed to the gap between the specimen and the roller at a feed rate of 0.75 kg·min^−1^. After each one-minute cycle, the specimen was cleaned by alcohol and weighed on an electronic balance of 0.1 mg accuracy to measure its weight loss. The total cycle number was ten. An average value of three tests was considered as the final result.

Dry-sliding tests were performed on a “Micron-tribo” tribometer (Micron-System, Kyiv, Ukraine) under a normal load of 5 N with back and forth reciprocating movements (each stroke was 3.5 mm long). Before dry-sliding wear testing, the surface of the specimens was prepared according to above standard metallographic procedure. Roughness parameters values were R_a_ = 0.076 μm, R_z_ = 0.409 μm for the coating and R_a_ = 0.106 μm, R_z_ = 0.934 μm for gray cast iron. The total sliding distance was 8.75 m (2500 strokes). The specimen served as a plate, whereas 100Cr6 steel (ISO 4957) balls of 3 mm diameter, SiC balls of 3 mm diameter, and diamond cones with an apex angle of 120° acted as counter-bodies. The dry-sliding behavior was evaluated by the coefficient of friction (CoF), and the volume loss for a wear track of 780 μm long was calculated by a “Micron-beta” profilometer (Micron-System, Kyiv, Ukraine) [49]. An average value of three dry-sliding tests was considered as the final result. As references, the specimens of gray cast iron were used in the initial (non-heat treated) state (further—the substrate) and after the same heat treatment as for the coating (further—the substrate-HT). The coating was wear-tested being in the heat-treated state.

## 3. Results

### 3.1. Microstructure and Phase Constituents

**Figure 1:**
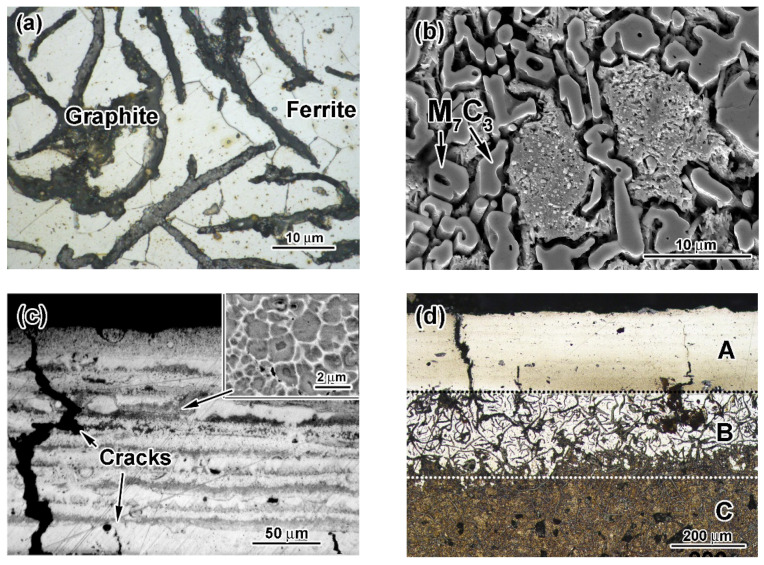

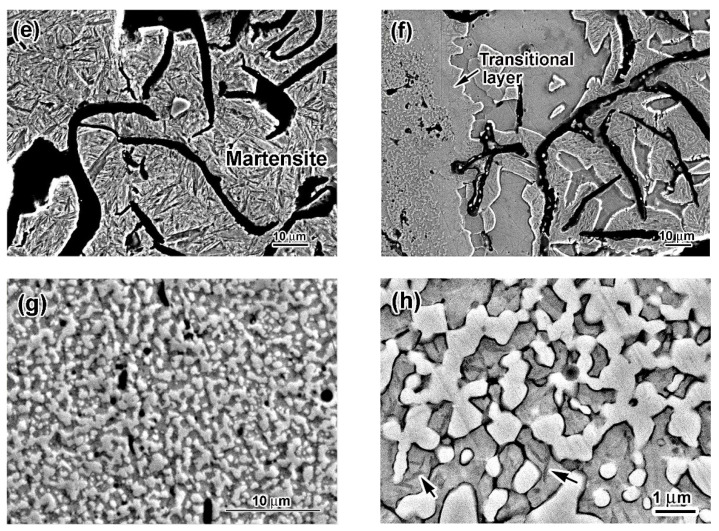
(**a**,**b**) Initial microstructures of gray cast iron and the 28 wt.%Cr cathode, respectively. (**c**) Microstructure of the as-deposited coating. (**d**) Near-surface structural zones in the heat-treated specimen. (**e**) Needle-shaped martensite and graphite lamellae in zone C. (**f**) Ferrite/martensite zone adjacent to the transitional layer. (**g**,**h**) Carbides in the coating.

The initial microstructures of the substrate and the cathode are displayed in Figure 1. The microstructure of the substrate (gray cast iron) consisted of ferrite grains with an average microhardness of 223 ± 25 HV and graphite lamellae of 0.5–3.0 μm thickness (Figure 1a). The microstructure of the cathode (hypoeutectic cast iron) was composed of solid-solution pre-eutectic dendrites surrounded by eutectic Cr-rich M_7_C_3_ carbides (Figure 1b) [42]. Hexagonal rod-shaped eutectic carbides with a volume fraction of 34–35 vol.% coalesced into agglomerates of 1–4 μm size.

**Figure 2:**
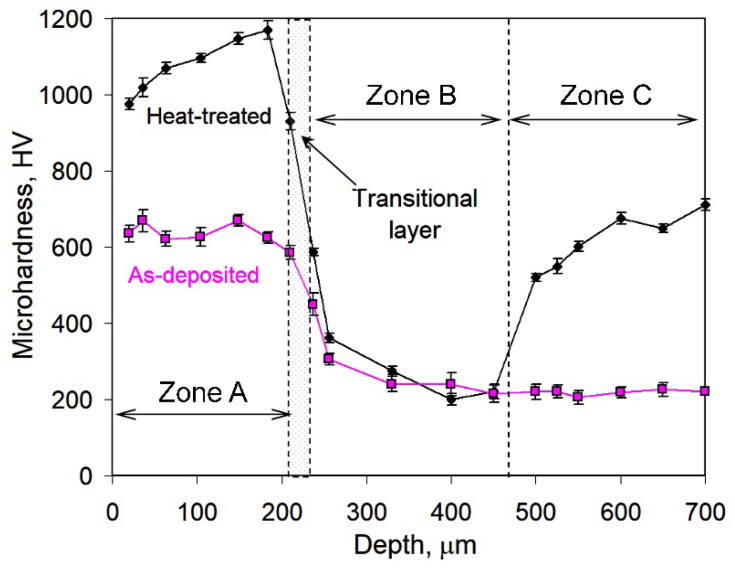
Cross-sectional microhardness profiles of the coated specimens (as-deposited and heat-treated).

A coating of 210–250 µm thickness was formed after pulsed-plasma deposition. The as-deposited coating possessed a laminar structure consisting of alternating bright and relatively dark bands (Figure 1c). High-magnification observation allowed revealing that these dark bands contained metal grains surrounded by a thin (0.03–0.12 µm) carbide network (insert of Figure 1c). Coarse eutectic carbides were not detected in the as-deposited coating structure. Moreover, cracks were seen in the coating to be oriented perpendicular to the coating surface. These cracks grew from the “coating/substrate” interface, and some of them went through the entire coating width. The coating contained pores with a volume fraction of 2.5 vol.%. The microhardness of the coating varied in the range of 620–670 HV (Figure 2).

After post-plasma heat treatment, a gradient pattern consisting of three clearly distinguished sub-surface zones (A (the coating), B, C) appeared in the near-surface layer (Figure 1d). Zone C was a bulk of the specimen with a microstructure consisting of graphite lamellae and needle-shaped martensite matrix having the microhardness of 553–710 HV (Figure 1e). Closer to the surface, martensite was gradually replaced by ferrite, forming a ferrite/martensite zone B of a variable thickness (190–290 µm) between the martensitic bulk and the coating. Ferrite was predominantly found in zone B, and martensite areas existed adjacent to graphite lamellae (Figure 1f). Numerous irregularly shaped carbide precipitates of 1 µm size coalesced into a broken network engulfing matrix grains (Figure 1g). Moreover, smaller roundish carbide precipitates (0.1–0.6 µm) were observed inside matrix grains (Figure 1h). A needle pattern was observed between carbide precipitates (shown by arrows in Figure 1h), revealing the formation of a high-carbon martensite matrix. The carbide volume fraction in the coating was calculated as 48.2 vol.%. Post-heat treatments increased the coating microhardness from 980 HV (near the top) to 1180 HV (close to the coating/substrate interface) (Figure 2). The microhardness profile had a recess from the ferrite–martensite matrix (zone B) to the martensite matrix (zone C). Zones A and B were separated by an 8–25 µm wide graphite-free transition layer containing a small amount of carbides (Figure 1f). The microhardness of the transition layer decreased from 930 HV at the coating boundary to 580 HV at the ferrite-martensite zone boundary.

**Figure 3:**
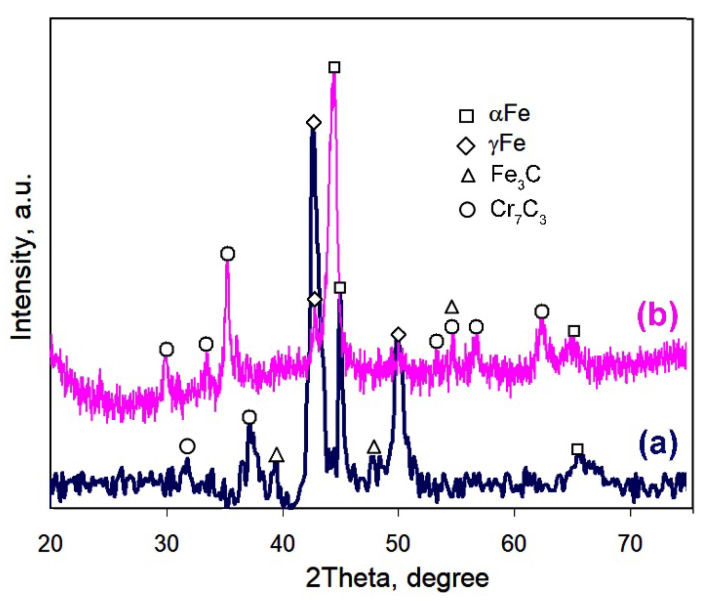
XRD patterns of the coating: (**a**) As-deposited state. (**b**) After post-heat treatment (αFe (6–0696), γFe (4–0829), (Cr,Fe)_7_C_3_ (5–0720), Fe_3_C (35–0772)).

The phase constituents of the coating were investigated by X-ray diffraction (Figure 3). The XRD pattern for the as-deposited coating contained peaks of matrix phases (γFe and αFe) and carbide phases (Cr_7_C_3_ and Fe_3_C). γFe (austenite) was found as the major phase, and its volume fraction in the matrix was 84 vol.%. Carbides were found in the matrix in a small amount according to their weak peaks. The XRD pattern of the post-heat-treated specimen contained the same set of peaks; however, a significant change in peak intensities was detected. The intensity of γFe drastically decreased, whereas the αFe peak had an increased intensity. The γFe→αFe phase transformation during post-heat treatment resulted in a retained austenite volume fraction (in the matrix) of 8.1 vol.%. Moreover, heat treatments led to an increase in the M_7_C_3_ carbide amount, as followed from numerous carbide peaks that appeared in the XRD pattern. Considering the total carbide volume fraction of 48.2 vol.%, the volume fractions of martensite and retained austenite in the heat-treated coating were recalculated as 47.6 vol.% and 4.2 vol.%, respectively.

### 3.2. EDS Characterization

**Figure 4:**
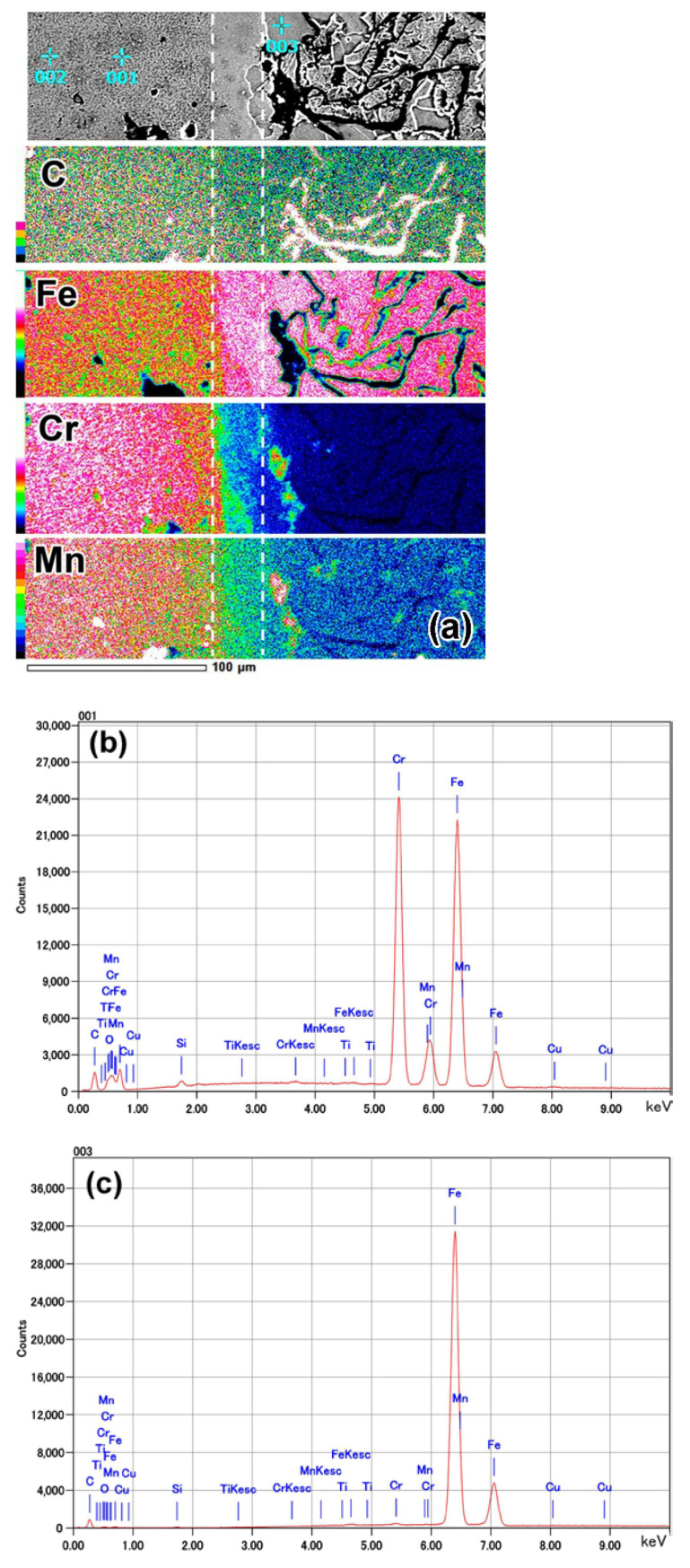

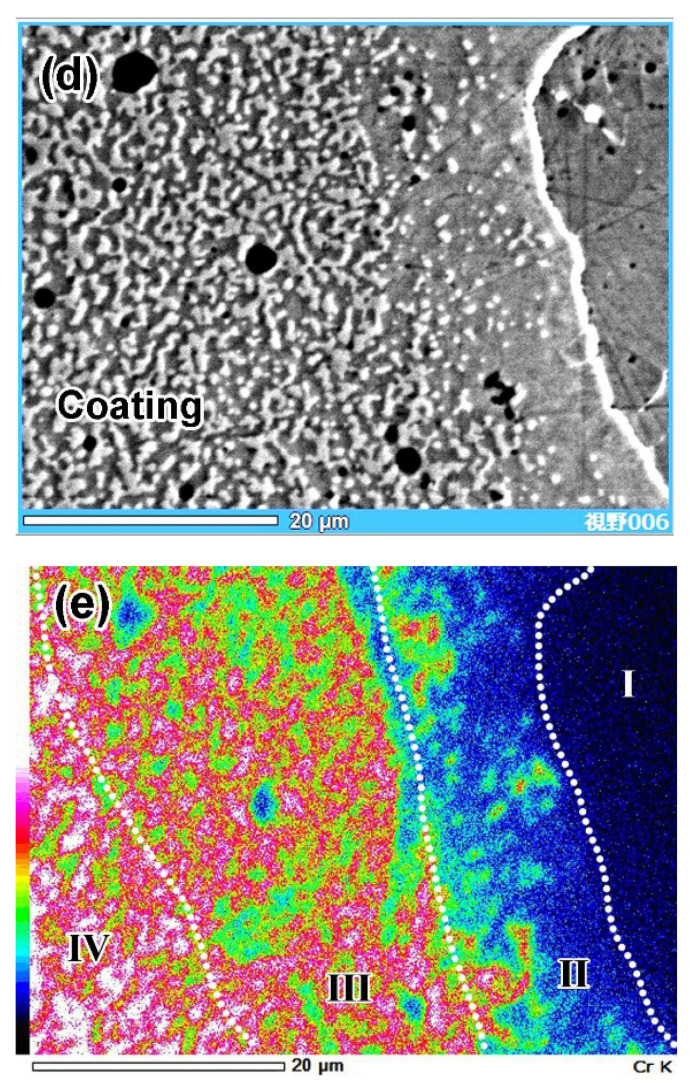
EDS analysis results: (**a**) Elemental mapping of C, Fe, Cr, and Mn. (**b**,**c**) EDS spectra of the local chemical analysis at points 001 and 003, respectively. (**d**) SEI image and (**e**) Cr mapping of the transition layer.

The EDS analysis was performed in a “mapping” mode to qualitatively assess the distribution of chemical elements in near-surface layers. The color of the “map” refers to elemental concentrations corresponding to the scale located on the left side of the “map” (from 0 wt.% (black) to 100 wt.% (white)). A gradient in elemental distribution with a gradual decrease in Cr, Mn, and C contents and an increase in the Fe content was noticed from the top of the coating to the substrate (Figure 4a).

**Figure 5:**
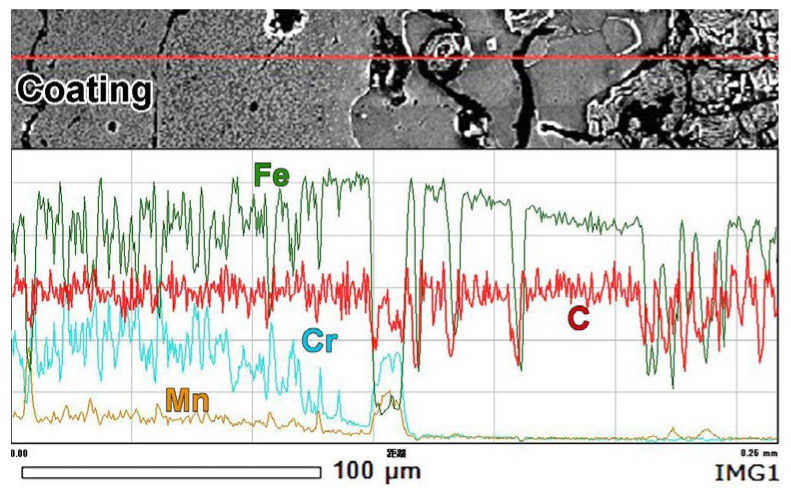
Cross-sectional EDS profile of Fe, Cr, Mn, and C from the coating to the substrate.

The Cr_Kα_ “mapping” more closely shown in Figure 4e reveals a smooth chromium gradient at the coating/substrate interface. Pores in the coating were enriched with manganese and chromium, reflecting the presence of oxides (MnO_2_, Cr_2_O_3_) in these pores. The transitional layer (area II) had slightly higher chromium content as compared to the base (area I). In the transitional layer, chromium was mainly accumulated in carbides, whereas the matrix was depleted in chromium. The coating had a much higher chromium concentration than the transitional layer; however, the distribution of chromium was uneven. The chromium concentration was low in area III and high in area IV, and it could be tracked by the color of carbides in areas III (pink) and IV (white-pink). The color of the matrix also changed from green in area III to pink-green in area IV. The different chromium contents in carbides of areas II, III, and IV reflected a variation of the carbide type from the transitional layer to the top of the coating. The “mapping” observation is supported by elemental profiles presented in Figure 5. The contents of chromium and manganese increased from the coating/substrate boundary to the top of the coating and became stabilized at a distance of 55–60 μm from the boundary.

The local EDS analysis of the coating at points 001 and 002 in Figure 4a reveals that the coating had a chemical composition of 29.43 wt.% Cr, 1.96 wt.% Mn, 0.62 wt.% Si, 0.68 wt.% Cu, and 0.03 wt.%Ti, indicating iron balance (Figure 4b). As the carbide size was less than the EDS spatial resolution [50], carbides and matrix areas both contributed to the EDS response. Therefore, the presented chemical composition could be considered as the average composition of the coating, and it corresponds to the composition of the cathode material (≈28 wt.% Cr cast iron). The ferritic matrix existing next to the transitional layer (point 003 in Figure 4a) contained 0.27 wt.% Cr, 0.88 wt.% Mn, 1.78 wt.% Si, and 0.09 wt.% Cu, indicating iron balance (Figure 4c). The presented EDS results should be considered as semi-quantitative because of a high EDS sensitivity for carbon contamination.

### 3.3. Tribological Characterictics

#### 3.3.1. Three-Body Abrasion Testing

**Figure 6:**
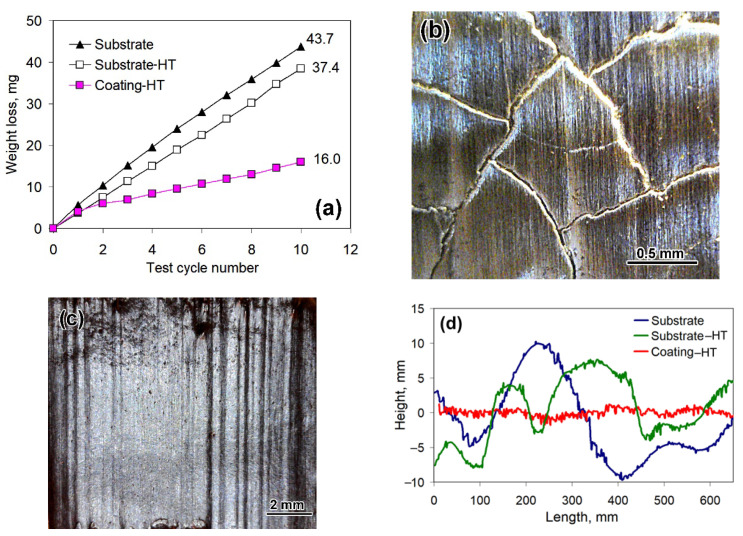
Abrasive wear characteristics of gray cast iron and the coating: (**a**) Cumulative weight loss curves. (**b**) Crack network on the abraded heat-treated coating surface. (**c**) Abraded substrate surface. (**d**) Worn surface profiles.

Figure 6 presents the results of “Three-body abrasion” tests. Upon completion of the tests, the total weight loss of the heat-treated coating (16.0 mg) was 2.7 times and 2.3 times lower than that of the substrate (43.7 mg) and the substrate-HT (37.4 mg) accordingly. During abrasive tests, all the specimens maintained close behavior only during the first test cycle when the coating roughness caused by plasma deposition was removed (Figure 6a), and afterward, the coating had a much lower wear rate (1.3 mg/min) than the substrate (4.2 mg/min) and the substrate-HT (3.7 mg/min). Evaluation under the stable wear process (from the second cycle to the 10th cycle) showed that the coating performed much better tribological behavior under abrasive conditions, which was 3.2 times and 3.0 times higher than the substrate and the substrate-HT.

The worn surface of the coating featured a closed crack network with a “cell” of 0.5–1.0 mm diameter. Inside the “cell”, the surface was smoothly abraded (Figure 6b) with the average roughness parameters of R_a_ = 0.635 μm and R_z_ = 2.184 μm (Figure 6d). The worn surface of the substrate had deep parallel grooves (Figure 6c), exhibiting a rougher profile with the average roughness parameters of R_a_ = 4.585 μm (increased by 7.3 times) and R_z_ = 9.033 μm (increased by 4.1 times). The worn surface of the substrate-HT had slightly less roughness with R_a_ = 3.436 μm and R_z_ = 8.573 μm, which was consistent with its lower weight loss compared with the substrate.

#### 3.3.2. Dry-Sliding Testing

**Figure 7:**
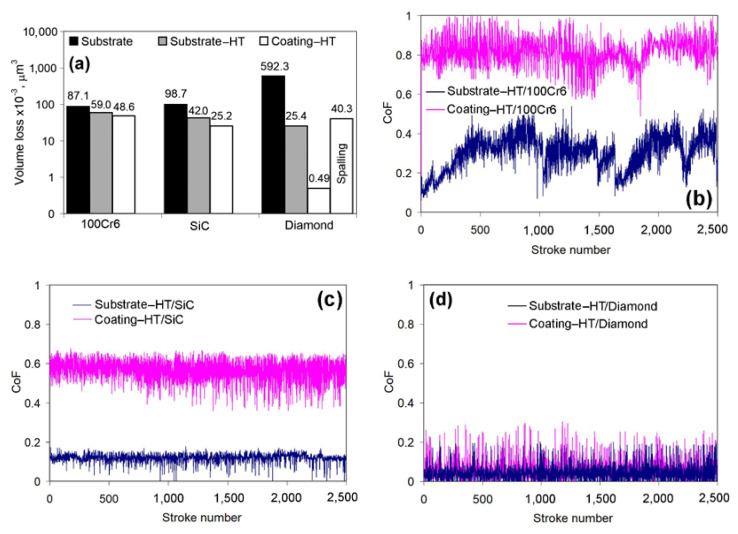
Dry-sliding wear characteristics of the substrate, the substrate-HT, and the heat-treated coating. (**a**) Consolidated data on specimens’ volume losses. (**b**) CoF variation for testing against 100Cr6 steel ball. (**c**) CoF variation for testing against SiC ball. (**d**) CoF variation for testing against diamond cone.

**Figure 8:**
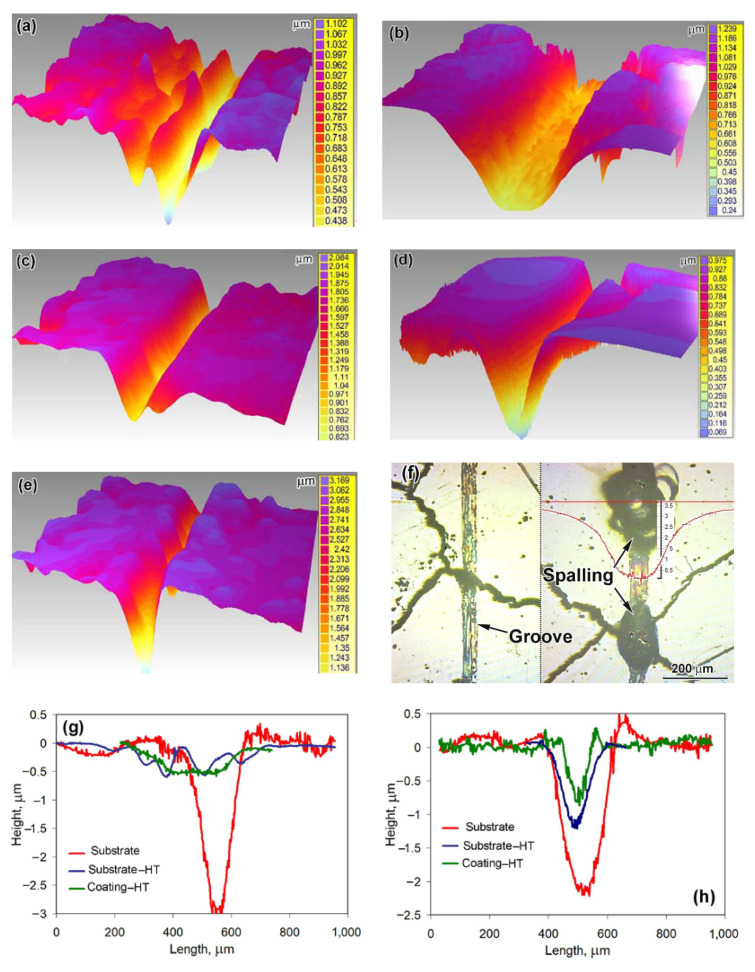

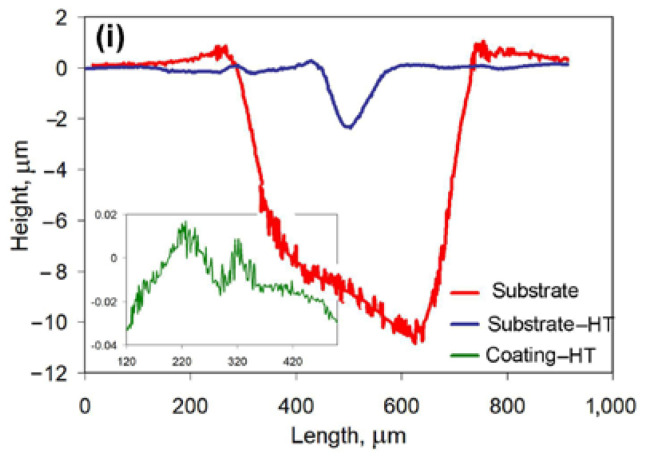
Dry-sliding worn surface characterization. Three-dimensional (3D) images of wear tracks on (**a**,**c**,**e**) the substrate-HT and on (**b**,**d**) the heat-treated coating after testing against (**a**,**b**) the 100Cr6 steel ball, (**c**,**d**) the SiC ball, and (**e**) the diamond cone. (**f**) Different fragments of a “diamond cone” wear track on the heat-treated coating (depth scale bar is given in micrometers). Wear track profiles for testing against (**g**) the 100Cr6 steel ball, (**h**) SiC ball, and (**i**) the diamond cone.

Figure 7 and Figure 8 present dry-sliding wear test results. Figure 7a presents the volume loss related to 1 μm of wear track. It is evident from Figure 7a that the volume loss of the substrate was in a direct proportion to the counter-body microhardness. The volume loss value slightly increased from 87.1 × 10^−3^ μm^3^ (against the steel ball) to 98.7 × 10^−3^ μm^3^ against the SiC ball and drastically increased to 592.3 × 10^−3^ μm^3^ against the diamond cone (Figure 7b–d). For the coating, the opposite tendency was found when its volume decreased from 48.6 × 10^−3^ μm^3^ (steel ball) to 0.49 × 10^−3^ μm^3^ (diamond cone) as the counter-body microhardness increased. The substrate-HT had intermediate volume loss values. The coating had a lower volume loss as compared to gray cast iron with any counter-body. The volume loss of the coating was 1.8 times, 3.9 times, and 1208.8 times lower than the substrate when it tested against the steel ball, the SiC ball, and the diamond cone, respectively. When compared to the substrate-HT, the advantage of the coating decreased: its volume losses were 1.2 times (the steel ball), 1.7 times (SiC ball), and 51.8 times (the diamond cone) lower than that of heat-treated GCI.

The CoF of the “Substrate-HT/100Cr6 ball” pair was characterized by pronounced instability with areas of increase up to 0.50–0.55 and a sharp decrease to 0.07–0.10 (Figure 7b).The CoF of the “Coating/100C6 ball” pair was more stable as compared to the previous one with a higher mean level (about 0.81) and a rather big scatter (0.62–1.00). Under sliding against the SiC ball, the CoF level decreased, while the difference in CoF remained approximately in the same proportion (Figure 7c). CoF values were rather stable with a decreased scatter: CoF smoothly fluctuated within 0.07–0.16 (mean value—0.12) for the substrate-HT and 0.35–0.67 (mean value—0.56) for the coating. Sliding against the diamond cone resulted in the lowest CoF level for both specimens. The “Substrate-HT/Diamond cone” pair performed CoF mean values of about 0.04 with a scatter of 0–0.20, while the “Coating/Diamond cone” showed a slightly higher CoF mean value (0.06) and a scatter (0–0.30) (Figure 7d).

Wear tracks observed on the worn surfaces were of different geometry depending on the specimen state and counter-body type, as seen from their 3D reconstruction (Figure 8a–e). The wear track profiles for testing against the steel ball are illustrated in Figure 8g. It is seen that wear tracks were wide and shallow (up to 0.5 μm) for the coating and the substrate-HT (the track for the latter was 1.5 times wider). In contrast, the substrate showed a deep (3 μm) and narrower (300 μm) wear track. When testing against the SiC ball (Figure 8h), all specimens performed the rather narrow wear grooves with a different depth: the width/depth of the groove were minimal for the coating (≈100 μm/ ≈1 μm accordingly) and maximal for the substrate. The most pronounced difference on the wear track profile was noted for testing against the diamond cone. As follows from Figure 8i, the wear track for the substrate was very wide and deep (up to 11 μm). The substrate-HT exhibited a narrow groove with moderate depth (2.1 μm). On the coating, the very shallow wear track of a negligible depth (0.015 μm) was formed (enlarged cross-sectional profile is shown in the insert of Figure 8i). The distinctive feature of a “diamond cone” wear track was the occasional spalling, which was observed mostly at the intersections of the groove and crack (Figure 8f right). The volume loss in spalling-free sections of a “diamond cone” wear track (Figure 8f left) was very low (0.49 × 10^−3^ μm^3^). However, the volume loss in spalling sections increased by two orders of magnitude (up to 40.3 × 10^−3^ μm^3^) due to the formation of deep (2–3 μm) holes by spalling.

## 4. Discussion

As shown above, pulsed-plasma deposition followed by post-plasma heat treatment significantly improved gray cast iron wear characteristics. GCI’s abrasion wear resistance increased by 3.0–3.2 times, while dry-sliding wear resistance increased by 1.2–1208.8 times, depending on the counter-body material. This happened due to the formation of a composite coating containing about 50 vol.% Cr-rich carbides in the structure [51].

The coating smoothly connected with the substrate through the transitional layer, indicating a strong metallurgical bonding between them. This phenomenon can be attributed to the melting of a GCI’s near-surface layer under the plasma flow. The possibility of plasma-induced surface melting could be evaluated by analyzing the temperature field of near-surface layers in contact with the plasma flow. The numerical modeling of substrate surface heating was executed based on the solution of a heat conduction task according to the approach of a short-term heat source (*q_o_*) evenly distributed over the target surface. The modeling approach was previously described in detail in [49]. Calculations were conducted using gray cast iron parameters: density = 7200 kg·m^−3^, specific melting heat = 140 kJ·kg^−1^. The temperature-dependent heat capacity and thermal conductivity values were adapted from the literature [52]. The value of specific power density (*q_o_* = 1.75 × 10^9^ W·m^−2^) corresponded to the EAPA working regime [43].

**Figure 9:**
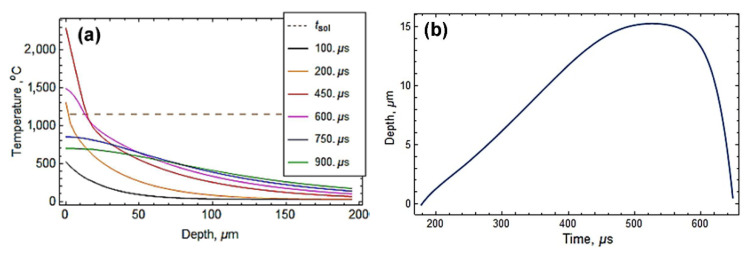
Numerical simulation results of the temperature field of the specimen under plasma heating. (**a**) Time-wise cross-sectional temperature distribution. (**b**) Time dependency of the melting depth (time is given from the contact with the plasma flow).

Numerical modeling results of the temperature distribution over the cross-section of the gray cast iron specimen are displayed in Figure 9a. It is noticeable that the plasma flow rapidly heated the substrate surface. After 200 μs of collision with the plasma flow, the substrate surface reached the solidus temperature (shown by the dashed line in Figure 9a) and melted to a depth of about 1.5 μm. Further heating resulted in an alteration of the melting depth according to the curve presented in Figure 9b. The surface temperature reached the maximum value of 2350 °C after 440 μs of the collision, whereas the melting depth reached the maximum value of 15 μm after 570 μs (the surface heating rate maximally reached 12.3 × 10^6^ K·s^−1^ [49]).During the next 100 μs of the collision, the substrate surface cooled below the melting temperature with the maximum cooling rate of 9.0 × 10^6^ K·s^−1^. The calculations showed the possibility of plasma-induced melting of the substrate, which is in accordance with a microstructure observation. The calculated melting depth (15 μm) complied with the transitional layer width (7–25 μm), confirming that the latter originated from surface melting. The molten layer of cast iron was mixed with the plasma liquid/gaseous material, forming a strong metallurgical coating/substrate bonding.

XRD analysis and microscopic observation results revealed that the as-deposited pulsed-plasma coating consisted mostly of austenite matrix (84.0 vol.%) with a small amount of M_7_C_3_ and M_3_C carbides. This observation confirms the previous findings for EAPA pulsed-plasma coatings deposited by a cathode made of eutectic Fe-based alloys (high-Cr cast iron and 18 wt.% W high-speed steel) [42,46]. The high-current discharge inside the EAPA chamber led to the evaporation and melting of the cathode [45,49]. Thus, the coating consisted of cathode erosion products, mostly microdroplets, that were transferred by the plasma flow to the target surface. These microdroplets, being deposited, underwent superfast cooling, which resulted in the inhibition of eutectic reactions [53]; thus, oversaturated (thermodynamically unstable) austenite grains were formed in the as-deposited coating. Austenite was partially decomposed through carbide precipitation under the heating induced by subsequent plasma pulses. M_7_C_3_ and M_3_C carbides precipitated in the form of a thin network around matrix grains (see the insert of Figure 1c). The formation of Cr-depleted cementite carbide was kinetically preferential at this stage [54].

The as-deposited structure is not suitable for abrasion applications because of its lower hardness. In order to improve the hardness and tribological behavior of the coating, post-plasma heat treatment was carried out [42,46]. During heat treatment, austenite grains turn into their thermodynamically stable state through the precipitation of Cr-rich carbides. The heat treatment at 950 °C for two hours allowed the diffusion of Cr atoms to form M_7_C_3_ carbide in the coating [55]. The precipitation of Cr-rich carbides led to austenite depletion by carbon and chromium enabling austenite→martensite transformation during oil cooling. The formation of hard phases (M_7_C_3_ carbides, martensite) increased the coating’s hardness up to 1180 HV. M_7_C_3_ carbides mainly precipitated as a network along grain boundaries. M_7_C_3_ carbides appeared on the cementite network, proving the transition of metastable M_3_C carbides into stable M_7_C_3_ ones. M_7_C_3_ carbides in the coating were four-fold smaller as compared to eutectic carbides in the cathode, confirming the refinement of the carbide phase under the plasma-assisted treatment [56].

During the prolonged holding at 950 °C, carbide precipitation was accompanied by the elemental partitioning in the coating and the substrate. Hence, the saturation of the substrate matrix with carbon occurred due to graphite dissolution, and then, carbon-rich austenite was transformed into high-carbon martensite, causing a significant increase of the matrix microhardness. Moreover, a counter element partitioning occurred: carbon was partitioned from the substrate to the coating, whereas chromium and manganese were partitioned from the coating to the substrate. Carbon diffused from the matrix toward the coating to bond with chromium to form carbides, thus making the matrix C-depleted, resulting in the formation of a ferrite–martensite zone (zone B) below the coating. On the contrary, Cr and Mn atoms diffused from the coating to the transitional layer in order to level their concentrations at the “coating/substrate” interface; hence, Cr distribution had a descending profile toward the substrate (Figure 5). The counter-flow of carbon and chromium atoms led to a structural gradient in the coating. Specifically, Cr depletion and C enrichment caused the appearance of layer III (Figure 4e), which contained Cr-depleted carbides (presumably, cementite (Fe, Cr, Mn)_3_C)). The matrix in layer III was also Cr-depleted, causing an increase of the *Ms* point and leading to a low austenite volume fraction (high martensite amount). Hence, layer III had the maximum microhardness (Figure 2), which reduced toward the surface due to an increase in the chromium content in the coating. These processes caused a smooth structural transition, contributing to the strong adhesion between the coating and the substrate.

The pulsed-plasma high-Cr coating showed an increased abrasive wear resistance due to the synergetic interaction of M_7_C_3_ carbides with martensite–austenite matrix, in which austenite grains might perform a TRIP effect under abrasion [7,57]. The volume fraction of carbides (about 50 vol.%) was notably higher than that of the EAPA cathode (28 wt.% Cr cast iron). The increase in the carbide volume fraction relative to the cathode was influenced by carbon enrichment of the coating. The sources of carbon enrichment could be (a) carbon atoms released due to the evaporation of bakelite walls of the EAPA channel under arc discharge [42] and (b) carbon atoms diffused to the coating from the substrate. Closely located carbides in the coating effectively resisted abrasion and led to shallow scratches with low surface roughness. On the contrary, the ferrite/graphite structure (substrate) had an increased wear rate with four to seven times higher roughness.

Dry-sliding wear tests have confirmed the enhanced tribological properties of the carbide coating. GCI had a lower CoF when it tested against steel and SiC balls due to the presence of a solid lubricant (graphite) in the structure [58]. As the coating did not contain graphite inclusions, its CoF increased by 2.7 times against the steel ball and by 2.0 times against the SiC ball as compared to the substrate. Despite this fact, the coating had much higher dry-sliding wear resistance, and its volume loss decreased by 1.8 times against the steel ball and by 3.9 times against the SiC ball as compared to the substrate. This phenomenon was caused by higher coating hardness (decreasing a depth of counter-body penetration) and by a low adhesion between coating carbides and the counter-body material (decreasing a galling manifesting). Notably, the coating had 1.9 times higher wear resistance against the hard SiC ball as compared to testing against softer steel balls. This fact can be explained by the much lower adhesion between the coating matrix (BCC/FCC lattices) and SiC (HCP lattice) related to the high hardness of counter surfaces and to a mismatch in their unit cell configuration. This assumption is also confirmed by the lower CoF (mean value of about 0.56) of the “coating/SiC” pair than that of the “coating/steel” pair (CoF = 0.81). Unexpectedly, the “coating/diamond cone” pair had a very low CoF (0.06) and negligible volume loss (0.49 × 10^−3^ μm^3^). Such CoF value indicates the minimal adhesion, which eliminated the galling-induced wear loss. The high hardness of the coating allowed mainly elastic strains in contact with the diamond cone, causing a small penetration to the coating surface and very shallow wear tracks. The wear process presumably proceeded through tribo-chemical interactions with oxide microchips spalling-off [59], which is accompanied by a negligible wear rate.

The high-Cr coating demonstrated a high tendency of crack formation during pulsed-plasma deposition. The merging of cracks led to a closed crack network, which covered the entire coating surface (Figure 6b). The most probable reason for cracking is the tensile stress generated in the coating [60] under its deformation (*ε*) upon cooling from the solidus temperature (*T_sol_*) to room temperature (RT):

(2) $$\sigma =\frac{\varepsilon \cdot E}{\left(1-\nu \right)},$$

where *E* is Young’s modulus and *ν* is Poisson’s ratio.

The total deformation can be considered as a sum of thermal contraction (*ε_T_*) and the linear effect of phase transformation (*ε_PT_*).

(3) $$\varepsilon =\varepsilon_{T}+\varepsilon_{PT}.$$

The constituents of Equation (3) can be roughly found as

(4) $$\varepsilon_{T}=\alpha_{A}(T_{sol}-Ms)+\alpha_{A}f_{A}(Ms-RT)+\alpha_{M}f_{M}(Ms-RT),$$

(5) $$\varepsilon_{P}{}_{T}=\frac{f_{M}\cdot \Delta V_{A\to M}}{3},$$

where *α_A_* and *α_M_* are the linear thermal expansion coefficients of austenite and martensite, respectively, *f_A_* and *f_M_* are the volume fractions of austenite and martensite, respectively, *M_s_* is the starting temperature of martensitic transformation, and Δ*V* is the volumetric effect of austenite→martensite transformation.

According to XRD analysis results, the as-deposited coating matrix contained 84 vol.% and 16 vol.% of the αFe phase. It can be assumed that αFe appeared from martensitic transformation; hence, the *M_s_* temperature could be derived from the Koistinen–Marburger equation.

(6) $$f_{M}=1-\exp(-a_{m}\cdot (M_{s}-TQ)),$$

where *a_m_* is a fitting coefficient (0.008 for Fe-1.86 vol.% C alloy [61]) and *TQ* is the quenching temperature (20 °C).

Now, taking *f_M_* = 16 vol.%, the *M_s_* temperature was calculated as 42 °C. The tensile stress was calculated according to Equations (2)–(6) using the following parameters: *T_sol_* = 1320 °C [49], *E* = 200 GPa [62], *ν* = 0.22 [63], *α_A_* = 18.6 × 10^−6^ K^−1^, and *α_M_* = 13.9 × 10^−6^ K^−1^ [64]. The volumetric effect of austenite→martensite transformation was derived from the equation: Δ*V_A_*_→*M*_ = 2.5–1.08·(C_γ_%) [65] assuming a carbon content in austenite (C_γ_%) of 1.50 wt.%. Eventually, the stress in the as-deposited coating was calculated as 5832 MPa, which is almost an order of magnitude higher than the ultimate tensile strength (UTS) of austenite. This stress was not relieved through plastic deformation because of superfast cooling after plasma heating; thus, it was relieved through the crack formation.

A similar cracking phenomenon was previously observed in a pulsed-plasma coating deposited using the cathode made of a high-speed steel of T1 AISI grade. The chemical composition of this steel (about 0.8 wt.% C, 18 wt.% W, 4 wt.% Cr, 1 wt.% V) was appropriate for the formation of a coating with fully austenitic matrix [46]. Completely different cracking behavior was demonstrated by the pulsed-plasma coating having a martensitic matrix [66].The martensitic coating was deposited in an EAPA chamber using a cathode of AISI 1020 low-carbon steel. Due to plasma-induced carbon enrichment [42], the coating possessed a high-carbon martensite structure (750 HV) with no cracks. Assuming *f_M_* = 90 vol.% and *f_A_* = 10 vol.%, the *M_s_* temperature was estimated as 310 °C (Equation(6)). Based on the above calculations, the stress value for martensitic coating was found as 2524 MPa, which is two-fold lower than that for austenitic matrix. The decrease in the stress was due to the compensation of thermal contraction (tensile stress) by the expansion caused by austenite→martensite transformation (compressive stress). The stress level was compared with the martensitic UTS which was derived from the correlation proposed in [67]:

UTS (MPa) = 3.734·HV − 99.8.(7)

Equation (7) was valid for the hardness range of 129–592 HV, and its extrapolation to 750 HV yielded a UTS value of 2701 MPa. Therefore, the stress calculated for the martensitic coating (2524 MPa) was lower than its UTS preventing crack formation. Concluding, the austenitic matrix is not appropriate for the pulsed-plasma coating, since it promotes crack formation due to its lower specific volume. By contrast, martensite has a much bigger specific volume (tetragonal lattice), contributing to crack prohibition. This issue should be considered when selecting a cathode material for coating deposition.

**Figure 10:**
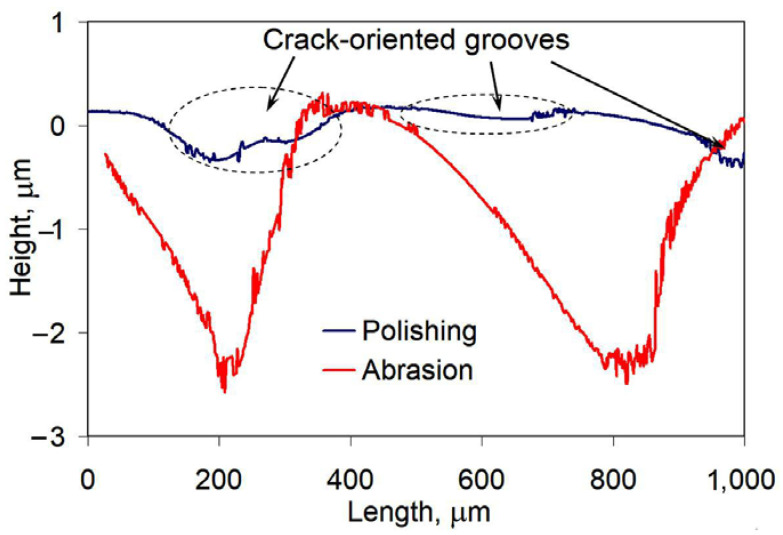
Surface profile of the coating after polishing and abrasive wear testing.

Cracking is a drawback of the studied high-Cr coating [68], since cracks accelerate abrasion at the crack edges. This resulted in a specific convex-shaped relief with the formation of grooves along crack edges and bulges between cracks. It is noticeable from Figure 10 that even after a smooth polishing, the grooves of about 0.5 μm depth appeared. During abrasive tests, localized wear led to increase in the grooves depth to about 2.5 μm. However, the surface roughness between cracks in convex-shaped areas was very low, reflecting an improved wear behavior of the coating. Thus, the coating abrasive response could be much higher if cracks are absent.

Moreover, the convex-shaped relief caused a “jumping” of the diamond cone that resulted in the surface impacting. Due to an increased coating hardness, the diamond cone penetrated slightly and caused high stress in the contact spot. Under the impact, the stress increased very fast and led to local brittleness and spalling of the coating. In contrast, dry-sliding against steel and SiC balls did not cause surface spalling, and it can be ascribed to lower stress in the contact spot of the “ball/plane” contact scheme.

The research showed the advantages and drawbacks of the plasma-deposited high-Cr coating. The coating has advanced wear behavior, and it is promising for the abrasive and dry-sliding applications when the local high-stress contacting is limited. An additional improvement of tribological abilities of the coating could be achieved by preventing cracking by means of obtaining the as-deposited martensitic matrix or by forming a composite structure in which the precipitates would decrease the thermal contraction of an austenite matrix. These approaches imply further intensive research. After overcoming the above shortcoming, the pulsed-plasma chromium carbide coating can be used to strengthen wear-loaded tool steels for cold stamping and forming of hybrid car bodies’, components of rolling equipment, die forms, etc.

## 5. Conclusions

The microstructure and wear characteristics of a pulsed-plasma coating reinforced with chromium carbibes under different wear conditions were evaluated. The following conclusions can be drawn:

1. A high-chromium coating of 210–250 mm width was fabricated on the surface of gray cast iron by atmospheric pulsed-plasma treatment using an eroded cathode made of high-Cr cast iron (2.3 wt.% C-27.4 wt.% Cr-3.1 wt.% Mn). The as-deposited coating had a γFe (84 vol.%)/αFe (16 vol.%) matrix with a low amount of carbides. Post-plasma heat treatment resulted in the precipitation of M_7_C_3_ carbides leading to the coating microhardness of 990–1180 HV and a microstructure consisting of 48 vol.% carbides (M_7_C_3_, M_3_C), 48 vol.% martensite, and 4 vol.% retained austenite.
2. Under pulsed-plasma deposition, the plasma-induced melting of the substrate took place, leading to a transitional layer of 8–25 µm width. The formation of the coating microstructure under post-plasma heat treatment occurred under counter-diffusion flow of Cr, Mn atoms from the coating to the substrate and the movement of C atoms toward them. This phenomenon led to a smooth elemental/structural gradient through low-carbide transition layer/ferrite/martensite zones, providing a good metallurgical bonding between the coating and the substrate.
3. The heat-treated coating performed the improved wear characteristics compared with gray cast iron (non-heat-treated and heat-treated conditions):
  - By 3.0–3.2 times higher abrasive wear resistance;
  - By 1.8–3.9 times lower volume loss (sliding against 100Cr6 steel ball)
  - By 1.2–1.7 times lower volume loss (sliding against SiC ball);
  - By 51.8–1208.8 times lower volume loss (sliding against a diamond cone).
4. The coating manifested a tendency of solidification cracking caused by tensile stress due to the formation of a mostly austenitic structure with a lower specific volume. Cracks locally facilitated abrasion wear and caused occasional spalling under sliding against the diamond cone. Under sliding against 100Cr6 steel ball and SiC ball cracks negligibly affected the coating wear mechanism.

## Data Availability

Data sharing is not applicable to this article.
